# Kallikreins 5, 6 and 10 Differentially Alter Pathophysiology and Overall Survival in an Ovarian Cancer Xenograft Model

**DOI:** 10.1371/journal.pone.0026075

**Published:** 2011-11-15

**Authors:** David Pépin, Zhong-Qi Shao, Geneviève Huppé, Andrea Wakefield, Chee-Wui Chu, Zahra Sharif, Barbara C. Vanderhyden

**Affiliations:** 1 Department of Cellular and Molecular Medicine, University of Ottawa, Ottawa, Ontario, Canada; 2 Centre for Cancer Therapeutics, Ottawa Health Research Institute, Ottawa, Ontario, Canada; 3 Ibex Pharmaceuticals Inc., Montreal, Quebec, Canada; The University of Kansas Medical Center, United States of America

## Abstract

Human tissue kallikreins (KLKs) are members of a multigene family of serine proteases aberrantly expressed in many cancer types. In ovarian cancer, 12 KLKs are upregulated, and of those KLK5, 6 and 10 have been the focus of investigations into new diagnostic and prognostic biomarkers. However, little is known about the contributions of KLK5, 6 and 10 to ovarian cancer pathophysiology.

In this study, a panel of 13 human ovarian cancer cell lines was screened by ELISA for secretion of KLK5, 6, 8, 10, 13, and 14. The ES-2 cell line, devoid of these kallikreins, was transfected with expression vectors of KLK5, 6 and 10 individually or in pairs. Co-expression of KLK5, 6 and 10 was correlated with lessened aggressivity of ovarian cancer cell lines as defined by reduced colony formation in soft agar and tumorigenicity in nude mice. ES-2 clones overexpressing KLK5, 10/5, 10/6, 5/6 made significantly fewer colonies in soft agar. When compared to control mice, survival of mice injected with ES-2 clones overexpressing KLK10, 10/5, 10/6, 5/6 was significantly longer, while KLK6 was shorter. All groups displaying a survival advantage also differed quantitatively and qualitatively in their presentation of ascites, with both a reduced incidence of ascites and an absence of cellular aggregates within those ascites. The survival advantage conferred by KLK10 overexpression could be recapitulated with the exogenous administration of a recombinant KLK10. In conclusion, these findings indicate that KLK5, 6 and 10 may modulate the progression of ovarian cancer, and interact together to alter tumour pathophysiology. Furthermore, results support the putative role of KLK10 as a tumour suppressor and suggest it may hold therapeutic potential in ovarian cancer.

## Introduction

The recently discovered tissue kallikreins are a family of secreted serine proteases encompassing 15 members (KLK1-15) whose genes (KLK1-15) are clustered in tandem on a 300 kb region on chromosome 19q13.4 [1]. KLK proteins are detected in many biological fluids including blood, seminal plasma, sweat, saliva, cerebrospinal fluid, milk, and interstitial spaces where they can be activated and/or inactivated by enzymatic cleavage [2]. KLKs cleave a broad range of substrates including extracellular matrix (ECM) proteins, insulin-like growth factor binding proteins, protease-activated receptors (PAR), other kallikreins and even themselves [2]. Moreover, KLKs are often expressed in groups, such as KLK3, 4, 5, 6, 8, 10, 13 and 14 in the breast or KLK2, 3, 4, 5, 11, and 15 in the prostate [2]. These observations have led to the hypothesis that kallikreins can act in a cascade to mediate their biological effects, also known as the KLK activome [3]. For example, preliminary evidence suggests that KLK5 may be an initiator of KLK cascades, capable of activating pro-KLK2, 3, 6, 7, 11, 12, 14, resulting in the degradation of ECM components of semen, and liquefaction [4].

Kallikreins have been implicated in a number of diseases such as Alzheimer's and multiple sclerosis [5], [6], inflammatory bowel disease [7], arthritis [8], sepsis [9], diabetes [10], skin diseases [11] and cancer [12]. Because KLKs are secreted and readily detectable in biological fluids, they have emerged as potentially valuable biomarkers, particularly in cancer, where KLK3 (also known as prostate specific antigen) has proven to be useful for prostate cancer monitoring. Most KLK expression is under hormonal control, and the responsiveness of KLK2 and 3 to androgens in prostate cancer cell lines [13], and KLK6 and 10 to estrogens in breast cancer cell lines is well documented [14], [15]. The pattern of expression of KLKs, as well as their hormonal regulation, suggests they may be involved in endocrine-related adenocarcinomas of the reproductive tract such as prostate, testis, breast, cervical, and ovarian cancers.

Accumulating evidence suggests that at least 12 of the 15 kallikreins are upregulated in ovarian cancer. Of those, KLK4, 5, 6, 7, 10, and 15 are associated with unfavorable prognosis while the expression of KLK8, 9, 11, 13, and 14 is associated with a favorable prognosis [12]. This study focuses on KLK5, 6 and 10 which are frequently overexpressed in ovarian cancer and found in elevated levels in the ascites and serum of patients [16]–[18]. Notably, serum KLK6 and KLK10 are indicators of poor prognosis [19], [20], and high KLK6 is associated with shorter recurrence-free survival and lower overall survival [21]. High levels of KLK10 in the serum are associated with advanced stage serous tumours with large residual disease and poor response to chemotherapy [22], while low levels of KLK10 in the tumour predict poor overall survival [23]. The histological subtypes of epithelial ovarian cancers, such as serous, mucinous, endometroid, clear cell and undifferentiated tumours may reflect distinct ontogenies and are becoming increasingly important in tailoring treatment [24]. The expression of KLKs is remarkably similar across histological subtypes. For example, all subtypes express KLK6, with perhaps a slightly higher proportion of clear cell tumours that display strong immunostaining for KLK6 [21], [25]. Similarly, patients with tumours of each subtype have detectable levels of KLK10 in their cytosols, with a slight but significantly higher proportion of KLK10 high patients being of the serous subtype [26]. Finally, KLK5 expression appears to be more prevalent in serous and undifferentiated tumours [27], [28].

While little is known about the biological basis for the contribution of KLK5, 6 and 10 to ovarian cancer, the ability of KLK5 and 6 to cleave ECM proteins [4], [29], and activate PAR signaling [30], suggest that they are directly implicated in various aspects of carcinogenesis. Degradation of ECM components may facilitate the detachment of malignant cells from the tumour and the invasion of normal tissues, while some of the released ECM peptides may have both pro and anti-angiogenic qualities [29], [31]. Moreover, PAR signaling has important roles in vasoregulation, cell growth and inflammation [32], [32,33]. KLK10 was identified as a putative tumour suppressor in breast [34] and gastric cancers [35], and is often silenced in ovarian cancer cell lines and tumours [36], despite its expression in the serum being an unfavorable prognostic marker. This apparent paradox exemplifies the dichotomy of kallikreins as both positive and negative regulators of processes involved in carcinogenesis such as angiogenesis, growth, invasion, and metastasis [37].

While evidence of aberrant expression of multiple kallikreins in ovarian cancer is mounting, little is known about their contribution to the pathophysiology of the disease. Herein we report the first attempt to unravel the contributions of KLK5, 6 and 10 in a xenograft model of ovarian cancer, and the first therapeutic use of a recombinant KLK10 protein *in vivo*.

## Materials and Methods

### Cell culture

The origin [38], and the type of tumours formed in xenografts [39] for the ovarian cancer cell lines Caov-3 (adenocacinoma [40]), OVCAR-3 (mildly differentiated serous adenocarcinoma [41]), OVCAR-4 (adenocarcinoma [42]), OV2008 (endometreoid adenocarcinoma with squamous differentation [39]), C13 (endometreoid adenocarcinoma with squamous differentation [39]), OVCA433 (adenocarcinoma [43]), SKOV-3 (clear cell adenocarcinoma [39]), OVCA429 (clear cell adenocarcinoma [39]), Hey (undifferentiated [39]), ES-2 (undifferentiated [39]), OCC-1 (undifferentiated [39]), A2780cp (undifferentiated [39]), and A2780s (undifferentiated [39]) used in this study as well as their culture conditions were described in a previous publication [39]. The cell lines HT1080 and NIH3T3, used as controls, were procured from ATCC (Manassas, VA, USA) and cultured according to their recommendations.

### Construction of stably transfected ES-2 cell lines over-expressing kallikreins

The plasmids pcDNA3.1D/V5-His/lacZ (Invitrogen, Mississauga, ON, Canada) with geneticin resistance, and pIRESpuro-2 (Clonetech, VWR, Mississauga, ON, Canada) with puromycin resistance were used as backbones and stably transfected into the ES-2 cell line to provide vector controls. In short, multiple clones stably transfected with pIRESpuro-2 were used as single vector controls, and multiple clones successively transfected by pcDNA3.1.1D/V5-His/lacZ and pIRESpuro-2 were used as double vector control. The cDNAs for KLK5, KLK6 and KLK10, as well as the pcDNA-KLK5 expression construct on a pcDNA3.1D/V5-His-TOPO backbone, were kindly provided by Dr. E.P. Diamandis (Toronto, ON, Canada). The KLK10 expression vector in pCMV-neo was provided by Goyal et al. and has been previously described [44]. Briefly, PCR amplification, restriction digestion and ligation of DNA fragments representing the cDNAs of KLK5, 6, and 10 into the expression vectors pIRESpuro-2 were performed, and the resulting constructs were stably transfected into ES-2 cells. A minimum of 3 clones of each were picked and one was randomly chosen to derive the respective cell lines ES-2-KLK5, ES-2-KLK6, and ES-2-KLK10 for *in vivo* experiments. For double transfectants, a minimum of 3 independent clones of pCMV-neo expressing KLK10 were further transfected with the pIRES-puro-2 expressing KLK5 or KLK6 and one of each was randomly chosen to generate respectively the ES-2-KLK5/10 and ES-2-KLK6/10 cell lines. The cell line ES-2-KLK5/6 was generated from one of the 3 clones by stably transfecting the ES-2-KLK6 cell line with the pcDNA-KLK5 construct. Transfection of ES-2 cells was carried out using Lipofectomine™ 2000 (Invitrogen, Mississauga, ON, Canada) according to the protocol provided by the manufacturer. The clones described above were selected and maintained in DMEM media (Thermo Scientific, Waltham MA, USA) containing geneticin (400 µg/ml) and/or puromycin (10 µg/ml) (Gibco BRL, Carlsbad, CA, USA).

### Cell Proliferation Assay

To evaluate proliferation, cell growth was analyzed in the parental ES-2 cells lines and 3 or more clones stably transfected with constructs for KLK5, KLK6, KLK10, KLK5/6, KLK5/10, KLK6/10 or Vector control using 12-well plates with initial plating densities of 10,000 cells/well. After 96 hours, cells were trypsinized and subsequently counted with a Coulter Counter (Beckman Coulter Inc., Fullerton CA, USA).

### Anchorage independent growth

The protocol used was previously described by M. Pace et al [45]. Briefly, 5×10^3^ cells were suspended in 3 ml complete media containing 3.5% low melting-point agarose and poured on top of the bottom layer of 7% agarose in the same medium in wells of a 6-well plate. Media (0.5 ml) was added to each well and changed every 2–3 days. A solution of *p*-iodonitrotetrazolium violet (1 ml) was added to each well at day 7 and colonies were stained for 24 hours, counted, and photographed.

### Invasion assay

For the invasion assay on the kallikrein overexpressing clones, we used the HTS transwell 96 system® (Corning, Lowell, MA). Briefly, The transwells were coated with basement membrane extracts as instructed by the manufacturer, and 5×10^4^ cells in 50 µl serum-free media were then added to the top chamber, while 150 µl of media with 10% serum were added to the reservoir. The plate was incubated at 37°C in 5% CO_2_ atmosphere for 24 hours. The cells which migrated to the bottom of the insert were stained with hematoxylin according to the manufacturer's protocol, and the membranes were mounted on slides, scanned using the ScanScope (Aperio, Vista, CA), and the amount of blue pixels was quantified using the Aperio software (Aperio, Vista, CA). Data was plotted as % invasion, when compared to the amount of cells on a control transwell membrane not coated with basement membrane extracts.

### Xenograft

All animal experiments performed in this study were in compliance with the *Guidelines for the Care and Use of Animals* established by the Canadian Council on Animal Care, and were approved by the Animal Care Committee at the University of Ottawa (Protocol ME-196). Female CD-1 nu/nu mice (Charles River Laboratory, Wilmington, MA, USA) aged 5–6 weeks were housed with food and water *ad libitum*, on a 12 h daylight cycle. The tumour cell IP xenograft method was described previously [39]. Briefly, after one week of acclimatization, the mice were injected intraperitoneally (IP) with 10^7^ ES-2 cells, or one of its derivative clones chosen at random from the cell lines stably transfected with KLK5, KLK6, KLK10, KLK5/6, KLK5/10, KLK6/10, Vector single control, or Vector double control, resuspended in 0.8 ml of phosphate-buffered saline. Groups were then blinded to ionvestigators until the end of the experiment at day 56. Disease progression was monitored daily, based on a set of general wellness criteria set by the animal care committee, and body mass was recorded twice a week until a predetermined endpoint was reached. The time at which symptoms of the disease first appeared, such as mild abdominal distension, or small palpable mass was recorded. Endpoints included: dehydration and/or weight loss of over 15% despite fluid therapy, any evidence of respiratory distress, body weight increase of over 5 g from the average body weight of control mice at the same age in the same population, presence of a palpable abdominal mass that impairs mobility, feeding or affects wellness and finally presence of abdominal distension that impairs mobility or affects wellness or causes significant discoloration evident on the dorsal or ventral skin.

Upon necropsy, tumour samples were weighed and divided to be either immediately flash-frozen in liquid nitrogen and stored at −20°C, or fixed in 10% buffered formalin (VWR, Mississauga, ON, Canada) for 24 hours and stored in 70% ethanol prior to processing into paraffin-embedded blocks, which were cut into 5 µm sections for hematoxylin and eosin (H&E) staining. Ascites volume was measured, and the samples were assessed microscopically to determine the presence of cellular aggregates. Samples were then spun at 2500× g for 10 minutes to collect the supernatant for storage at −20°C for subsequent measurements of KLK levels by ELISA.

### Blood sampling

For the survival experiment, blood samples were taken one week prior to injection and weekly until either the animal reached endpoint or the experimental period ended at day 56. Blood was also taken before necropsy whenever possible. For the recombinant KLK10 blood clearance and toxicity experiment, each dosage group (0, 0.2, 1, and 5 mg) had one animal per timepoint (−1 h, 1 h, 2 h, 4 h, 6 h, 8 h, 12 h, and 24 h). To recover the plasma, 100–200 µl of blood was acquired by saphenous vein puncture with a 25G5/8 gauge needle (BD, Franklin Lakes, NJ, USA) and collected into microvettes® CB300LH (Sarstedt, Germany) coated with heparin, centrifuged 5 min at 2000 g, and the plasma layer was separated to be stored at −20°C until the ELISAs were performed.

### ELISA of kallikreins

For the panel of ovarian cancer cell lines, cells were cultured in 24-well plates with 5×10^4^ cells and 1 ml of media per well. Media samples were collected after incubation at 37°C for 3 days. ELISAs for KLK5 [46], KLK6 [47], KLK8 [48], KLK10 [49], KLK13 [50], and KLK14 [51] were performed according to the protocols published previously. ELISAs for the KLK5, 6 and 10 were performed on media of ES-2 clones after 24 h of culture on the day of xenograft implantation. Similarly, ELISA were performed on both human ascites samples and mouse serum and ascites samples diluted from 5-fold to 8000-fold, depending on the KLK concentration, in a dilution buffer (50 mM Tris-Cl pH7.8, with 60 mg/ml of BSA and 0.5 mg/ml of sodium azide).

### Recombinant KLK10 production

KLK10 cDNA was amplified by PCR using oligos KLK10FP (TATACGTAGCGCTGCTCCCCCAAAACGACAC) and KLK10RP (GTCCTAGGATCGATTGGAGCGTATGAC) [44] from a pCMV-neo vector carrying KLK10 cDNA. After double digestion with *SnaB*I and *Avr*II, the amplified DNA fragment was inserted into pPIC-9, pre-digested with *SnaB*I and *Avr*II. The resulting plasmid, pPIC-KLK10, was then transformed into the *Pichia pastoris* host strain KM71 by electroporation (*Pichia* Expression kit, Invitrogen life technologies).

Fermentation of 15-litres of recombinant KLK10 was conducted using a BIOSTAT ® ED fermenter (B.Braun Biotech International, Allentown, PA, USA) and a process based on Pichia fermentation Process Guidelines from Invitrogen. Briefly, fermenter was inoculated with yeast prepared in a 2800 ml shaker-flask for a starting OD_600_ of ∼0.3. After a 20-hour glycerol batch phase, a 4-hour glycerol feed phase was followed. Induction was initiated by starting glycerol feeding and lasted for about 40 hours. Cells were removed by centrifugation and supernatant was collected.

Purification of KLK10 from the supernatant was carried out using a CM-sepharose column (Amersham Biosciences, ON, Canada) as described previously [49].

### Treatment with recombinant KLK10

For the blood clearance experiment, we first tested single bolus IP doses of recombinant KLK10 (0, 0.2, 1, and 5 mg in 1 ml) with 5 nu/nu mice per dose and sampled the blood at various time points as described above. The animals were closely monitored for the first 12 h, and then periodically for 15 days before being sacrificed.

For the toxicity experiment we tested doses of 0, 50, 200, and 800 µg in 1 ml of KLK10, administered daily IP in 3 animals per group for 7 days, followed by 7 days of daily monitoring with no treatment before being sacrificed. At necropsy, the liver, lung, heart and kidney were removed and divided to be either immediately flash-frozen and stored at −20°C or fixed in 10% buffered formalin (VWR, Mississauga, ON, Canada) for 24 hours and stored in 70% ethanol prior to processing into paraffin-embedded blocks, which were cut into 5 µm sections for H&E staining. Sections were analyzed for signs of inflammation and damage.

For the therapeutic experiment, the nude mice were randomly divided into one control and 2 treatment groups (8 animals/group). Treatment duration was 14 days and the study ended at 8 weeks post-xenograft. Animals still alive at the end of the study were sacrificed. On day 1, animals were injected IP with 0.2 ml of PBS buffer, or 0.2 ml of PBS containing 5 mg of KLK10 followed immediately by an injection of 10^7^ ES-2 cells resuspended in 0.8 mL PBS buffer. From then on, 1 ml of PBS buffer or 1 ml of PBS containing 5 mg of KLK10 were injected IP to each animal either daily or twice daily (as indicated) from day 2 to day 14.

For the *in vitro* treatment experiment, 10^5^ ES-2 cells per well were seeded in a 12-well plate containing either serum-free DMEM or DMEM with 10% fetal calf serum, and supplemented with 4 doses or recombinant KLK10 (0, 300 ng/ml, 3000 ng/ml and 30000 ng/ml) for 96 h. Cell viability was determined by trypan blue exclusion and cells were counted using a ViCell Counter (Beckman Coulter, Fullerton, CA).

### Survival curves and statistical analyses

Kaplan-Meier survival curves were plotted using GraphPad Prism 4.0 software (Graphpad Software, San Diego, CA, USA) and compared using a logrank test. Pathophysiological parameters such as ascites volume and tumour burden (total tumour mass excised) and results from *in vitro* experiments were compared by one-way ANOVA followed by Tukey's post test. Proportions such as incidence of aggregates or ascites were compared by CHI square. Statistical significance was inferred at p<0.05.

## Results

### Secretion of kallikreins 5, 6 and 10 correlates with reduced aggressiveness in a panel of ovarian cancer cell lines, yet is detectable in the ascites of ovarian cancer patients

Expression of the kallikrein cluster including KLK4 to KLK14 has previously been reported in ovarian cancer [37]. However it has also been reported that different kallikreins can have diametrically opposed effects on patient prognosis in a variety of cancers [37]. To verify that kallikrein expression is recapitulated in ovarian cancer cell lines, a panel of thirteen ovarian cancer cell lines (CAOV-3, OVCAR-3, OVCAR-4, OV2008, C13, OVCA433, SKOV-3, OVCA429, Hey, ES-2, OCC-1, A2780cp, A2780s) was screened for secretion of KLK 5, 6, 8, 10, 13 and 14 into the culture media by ELISA (Table S1). On the basis of kallikrein expression, the cell lines could be segregated into non-expressors (SKOV-3, OVCA429, Hey, ES-2, OCC-1, A2780cp, A2780s) and expressors (CAOV-3, OVCAR-3, OVCAR-4, OV2008, C13, OVCA433). In the latter group, all shared common expression of KLK5/6, and 5 of 6 expressed KLK10, 4 of 6 KLK8, 3 of 6 KLK13 and none expressed KLK14. To investigate any link between kallikrein expression and aggressiveness of the cell lines, these two groups were compared for their ability to invade into matrigel, form colonies in soft agar and develop tumours intraperitoneally in nude mice (data not shown). A loose correlation was observed, albeit with some exceptions, that in contrast to the non-expressors, the cells expressing kallikreins did not invade matrigel, did not form colonies in soft agar, and as previously reported by us [39], were very poor at forming tumours in nude mice (Table 1).

**Table 1:** Kallikrein expression profile and tumorigenicity in a panel of 13 ovarian cancer cell lines.

Cell lines	KLK secretion (in media)	Invasion (matrigel)	Colony formation(agarose)	Xenografts (mice) [39]	
				Tumour incidence	Median Survival (days)
CAOV-3	5,6,8,10	−	−	Unknown	N/A
OVCAR-3	5,6,8,10	−	−	0/6	N/A
OVCAR-4	5,6,8,10,13	−	−	Unknown	N/A
OV2008	5,6,8,10,13	−	−	4/5	66
C13	5,6,10,13	−	−	0/5	N/A
OVCA433	5,6	−	−	0/3	N/A
SKOV-3	ND	+/−	+/−	2/7	105
OVCA429	ND	+	+	3/3	62
HEY	ND	+	+	2/3	24
ES-2	ND	+	+	5/5	16
OCC-1	ND	−	+	3/3	15
A2780cp	ND	−	+	3/3	24
A2780s	ND	−	+	3/3	46

ND = not detectable. N/A = Not applicable.

### Stable overexpression of KLK 5, 6 and 10, alone or in pairs, in clones of the kallikrein-deficient ES-2 cell line, results in altered anchorage-independent growth but does not affect cellular proliferation or invasive potential

**Figure 1:**
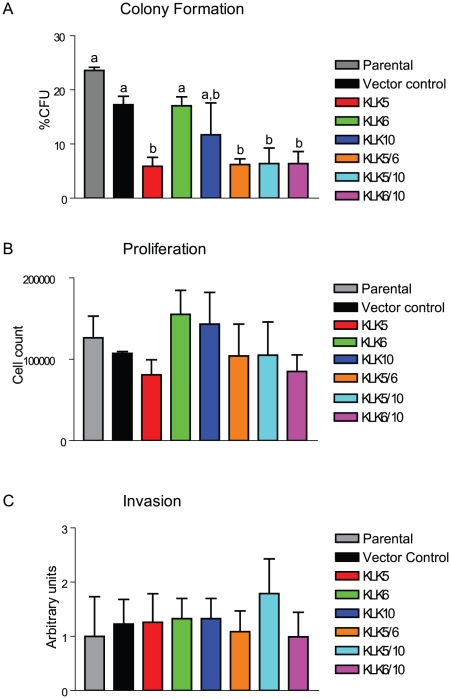
Clones overexpressing KLK5, 6 and 10, alone or in pairs, display differential anchorage-independent growth but do not differ in proliferation or invasive capacity. Three or more clones of the ES-2 cell line overexpressing KLK5, 6 or 10 or pairs of KLK5/6, KLK5/10 and KLK6/10 were compared to parental ES-2 cells or vector-transfected controls *in vitro* for their tumourigenic potential. A) Clones were grown in soft agar and the number of colonies was counted and is represented as percentage of the cells which formed colonies. B) Clones were grown for 96 h in serum-containing media and cell numbers were counted. C) Clonal cells resuspended in serum-free media were deposited in an insert coated with basement membrane extract and allowed to invade the transwell bathing in media with 10% serum for 24 h and migrating cells were quantified. The results are shown as the mean of 3 or more clones +/− SEM, and significance is inferred by one-way ANOVA with post test if p<0.05. Different lower-case letters above each bar indicate statistically significant differences between values (p<0.05). In C, the data are normalized to the parental control.

KLK5, 6 and 10 were the most commonly expressed kallikreins in the less aggressive ovarian cancer cell lines suggesting a correlation between the expression of those kallikreins and tumourigenic potential. To tease apart the roles of each kallikrein and their interactions on tumourigenicity, ES-2 cells which do not express any of the tested kallikreins (Table 1) were stably transfected with expression vectors for KLK5, 6, 10 alone or in pairs. 3 or more clones of KLK5, 6, 10, 5/6, 5/10, 6/10 along with empty plasmid control and unmodified ES-2 cells were compared for anchorage-independent growth, proliferation and invasion (Fig. 1). Expression of KLK5, 5/6, 5/10, and 6/10 was sufficient to significantly reduce the ability of ES-2 cells to form colonies in soft agar when compared to vector-only control, but did not alter the rate of proliferation over 96 h or modulate the ability of the clones to invade in a transwell assay.

### Stable overexpression of KLK 5, 6 and 10, alone or in pairs, in clones of the kallikrein-deficient ES-2 cell line, results in altered survival of a mouse xenograft model

**Figure 2:**
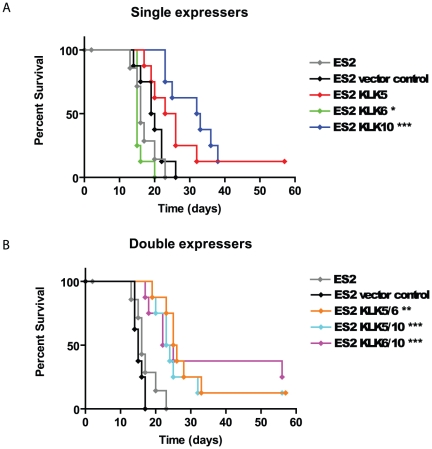
Clones overexpressing of KLK5, 6 and 10, alone or in pairs, differentially affect survival times when xenografted into nude mice. A) Clones of the ES-2 cell line overexpressing KLK5, 6 or 10 or B) pairs of KLK5/6, KLK5/10 and KLK6/10 were injected IP in nude mice and survival was compared to control mice xenografted with the appropriate vector backbone clones. The results are shown as a Kaplan-Meier plot, and significance using a logrank test versus appropriate control was inferred at p<0.05. * denotes p<0.05, ** denotes p<0.01, and *** p<0.001.

To investigate whether differential kallikrein expression could regulate the aggressiveness of ovarian cancer cells *in vivo*, an intra-peritoneal (IP) xenograft model was employed. For this purpose the ES-2 ovarian cancer cell line is ideal, since it does not express kallikreins 5, 6 and 10 (Table 1) and readily forms rapidly-progressing tumours IP in nude mice that are accompanied by ascites, thus mimicking disease progression in humans [39]. Clones derived from this cell line, stably secreting KLK5, 6, 10, 5/6, 5/10 and 6/10 in the culture media along with the appropriate empty vector controls (Table 2) were injected IP in immunodeficient nu/nu mice. The mice were injected with 10^7^ cells of each clone per animal, in groups of 8, which were then blinded, and closely monitored for endpoints. Median survival times for the parental line was 16d whereas the single expressor control was 19.5d and the double expressor control was 15d. Survival of the group expressing KLK5 (24.5d) did not differ from the control group (Fig. 2), while survival of the groups expressing KLK10 (32.5d), KLK5/6 (25.5), KLK5/10 (23.5), and KLK6/10 (23.5) was significantly longer than their appropriate controls by logrank test (Fig. 2). Survival of the group overexpressing KLK6 (15d) alone was significantly shorter than the control cell line but not the parental line. The groups KLK5, KLK10, KLK5/6 and KLK5/10 each had one disease-free surviving mouse, while group KLK6/10 had two, upon study termination at day 56, whereas all the mice in the control groups developed disease.

**Table 2:** Stable overexpression of KLK5, 6, and 10 alone or in pairs, in clones of the ES-2 cell line results in secretion of kallikreins into the culture media.

ES-2 clone	Kallikrein concentration in media (ng/ml)		
	KLK5	KLK6	KLK10
Parental	<0.2	<0.2	<0.5
Single vector control	<0.2	<0.2	<0.5
Double vector control	<0.2	<0.2	<0.5
KLK5	227	<0.2	<0.5
KLK6	<0.2	352	<0.5
KLK10	<0.2	<0.2	316
KLK5/6	58	202	<0.5
KLK5/10	74	<0.2	40
KLK6/10	<0.2	260	1400

### Mice xenografted with kallikrein-secreting tumours display changes in pathophysiology

To clarify the link between KLK secretion and survival, several disease-related metrics were compared across all groups upon necropsy (Table 3). The most prevalent endpoint was abdominal distension (82%) resulting from ascites accumulation, followed by respiratory distress (8%) caused by pleural effusions, dehydration and weight loss (7%), and finally impaired mobility (3%). Some animals were not measured in the endpoint statistics because of the absence of disease upon study termination (N = 8), or because they died of the disease prior to being endpointed (N = 6). Tumour histology, spread and sites of metastases were similar amongst groups, with a preference for the omentum, peritoneal membrane, diaphragm, reproductive organs, liver and intestines. Both tumour burden and ascites volume were recorded in animals who reached endpoint, and non-zero values were used to calculate the mean (Table 3). Mean ascites volume did not differ between groups with the notable exception of control-double which progressed past their distension endpoint before being sacrificed because of their rapid rate of disease progression. A statistically significant lowered incidence of ascites at necropsy was observed in animals of groups KLK5/10 (p<0.01) and KLK6/10 (p<0.01) with only 37.5% occurrence rate when compared to control groups which all developed ascites. Paradoxically, the KLK6/10 group also had on average a higher tumour burden (p<0.01), likely because of the longer ascites-free survival. Amongst the animals that did develop ascites within the groups of KLK5, KLK10, KLK5/6, KLK5/10, KLK6/10, a significantly lower incidence of multicellular free-floating aggregates in the ascites was recorded (Table 3). The aggregates present in the ascites were compact spheres of cells of uniform size (∼1 mm^3^) visible to the naked eye. The kallikrein concentrations measured by ELISA in the ascites showed levels of KLK6 (Table 3) to be comparable to levels seen in patient ascites, while levels of both KLK10 and KLK5 were elevated in comparison to the patient samples, especially in the combination groups.

**Table 3:** Nude mice xenografted with ES-2 derived clones overexpressing KLK5, 6 and 10 alone or in pairs, develop different pathophysiologies.

Groups	N	Ascites incidence (%)	Mean ascites volume (ml)	Aggregates (%)	Mean KLK concentration in ascites (ng/ml)	Mean tumour burden (g)	Median KLK detection onset (days)	Median symptom onset (days)
Parental	7	100	3.97±0.52	85.7	ND	0.72±0.16	N/A	13
Single vector	8	87.5	2.57±0.79	85.7	ND	1.34±0.19	N/A	16.5
KLK5	8	75	3.70±0.58	33.3	905±352	1.65±0.17	12	21
KLK6	8	87.5	3.97±0.51	71.4	522	1.15±0.18	5	14
KLK10	8	87.5	3.86±0.40	0*	3680±850	1.53±0.16	19.5	24.5
Double vector	8	100	8.23±0.38	100	ND	1.40±0.15	N/A	13
KLK5/6	8	75	4.25±0.73	0*	KLK5 1890±836KLK6 1290±513	1.26±0.16	8	20
KLK5/10	8	37.5*	3.52±0.87	0*	KLK5 393±112KLK10 464±301	2.34±0.23	14	21
KLK6/10	8	37.5*	4.00±1.58	0*	KLK6 1612±684KLK10 16618±1230	3.97±0.42	7	16.5

ND = not detectable; N/A = not applicable;

*denotes statistical significance relative to appropriate control at p<0.05 by CHI-square or one-way ANOVA where applicable. Mean values are indicated ± SEM.

**Figure 3:**
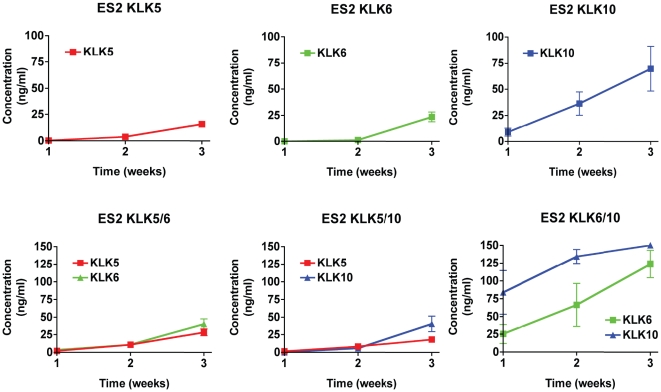
Plasma kallikrein levels reflect the progression of the disease in xenografted mice. Plasma kallikrein levels were recorded weekly by ELISA in the mice xenografted with ES-2 clones overexpressing of KLK5, 6 and 10, alone or in pairs. The last three weekly measurements before endpoint of individual mice were plotted as the mean concentration of the group +/− SEM.

The survival time of each group of mice can be divided into a period prior to onset of symptoms, followed by a symptomatic period which culminates at endpoint. Variability between groups is already present when looking at the onset of symptoms (Table 3), suggesting kallikreins may affect early disease progression. To follow the early disease progression, plasma kallikrein levels were measured by ELISA in each animal weekly and upon necropsy, to serve as a surrogate marker of tumour burden. Kallikreins were detectable in the plasma well before the onset of the first symptoms in all mice, suggesting that asymptomatic trace disease is detectable by measuring circulating kallikreins. In all groups, serum concentration quickly rises and culminates with endpoint as seen in the last three measurements (Fig. 3). Additionally, the surviving tumour-free animals of groups KLK5/6, KLK5/10, and KLK6/10 (Fig. 2B) did not display any detectable levels of kallikreins in the plasma for the duration of the study.

### Intraperitoneal administration of recombinant KLK10 recapitulates increased survival in an ES-2 xenograft model

**Figure 4:**
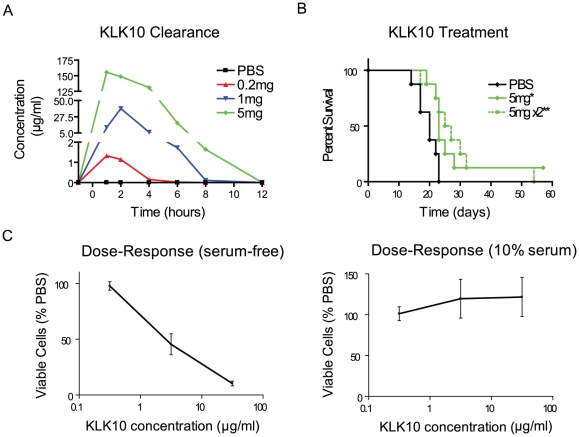
Treatment of mice xenografted with ES-2 cells with various IP doses of recombinant KLK10. A) Mice were injected with a bolus of recombinant KLK10 IP and blood samples were taken at different time intervals to measure plasma concentrations of KLK10 by ELISA. B) Mice were injected IP with either PBS or recombinant KLK10 daily or twice daily for 14 days post xenograft with ES-2 in a survival experiment. C) ES-2 cells were treated with various doses of recombinant KLK10 (0, 0.3, 3, 30 µg/ml) for 96 h in serum-free or serum-containing media and cell viability was determined by trypan blue exclusion. * denotes p<0.05, ** denotes p<0.01, and *** p<0.001.

To further confirm that the observed anti-tumourigenic effects of kallikreins were specific, a survival experiment using recombinant KLK10 was performed since, as a single agent, it showed the most promise (Fig. 2A). A pilot study was first conducted to ensure the recombinant KLK10 had no side effects in healthy mice before testing it in tumour-bearing animals. A single bolus IP dose (0, 0.2, 1, 5 mg) of KLK10 or daily IP injections (0, 0.05, 0.2, 0.8 mg) for 14 consecutive days were both well tolerated with no changes in body mass or general wellness, and no visible toxicity upon review of tissue sections of the liver, lung, heart and kidney (data not shown). The drug was judged safe and suitable for treatment of tumour-bearing animals, with doses of up to 5 mg being completely cleared of the blood by 12 h (Fig. 4A).

To test the efficacy of the recombinant KLK10, IP doses of 5 mg were administered once or twice daily for 14 days and compared to PBS injected control in the ES-2 xenograft model. Statistically significant increases in survival were observed in animals treated with recombinant KLK10 at 5 mg once daily (p<0.05), and twice daily (p<0.01), and one complete responder was found to be disease-free at the end of the study (Fig. 4B). Tumour burden and ascites volume did not differ from control in treated mice (Fig. S1), and disease presentation such as aggregates in ascites or sites of metastasis was not qualitatively different (data not shown).

The recombinant KLK10 doses injected IP lead to plasma concentrations orders of magnitude higher at one hour (Fig. 4A) than the highest doses recorded in KLK10 tumour-bearing mice (Table 3), albeit transiently. To investigate if such doses could be cytotoxic, ES-2 cells were treated *in vitro* with increasing concentrations of recombinant KLK10 for 96 h in the presence or absence of serum. Recombinant KLK10 caused significant cell death when compared to PBS-treated control, although this effect was completely inhibited by adding 10% serum to the culture media (Fig. 4C).

## Discussion

This study revealed for the first time a correlation between expression of multiple kallikreins (KLK5, 6, 8, 10, 13 and 14) and reduced aggressivity in a panel of 13 ovarian cancer cell lines. Of the kallikreins tested, KLK5, 6 and 10, were the most consistently expressed in cell lines with a less aggressive phenotype, which were incapable of forming colonies in soft agar, invading matrigel or forming tumours in nude mice. Paradoxically, KLK5, 6 and 10 are expressed in a high proportion of ascites of ovarian cancer patients [52], and they have previously been associated with poor patient prognosis in ovarian cancer [37]. KLK5, 6 and 10 are detectable in the ascites of ovarian cancer patients at the relatively high average concentrations of 62.2 ng/ml, 144 ng/mL, and 57 ng/ml respectively [16], however few studies have addressed the differences in ascites and serum concentrations of kallikreins on the basis of histological subtypes in a large cohort of ovarian cancer patients. Furthermore, the role of kallikreins in ovarian cancer progression has been scarcely studied outside of prognostic and diagnostic applications, and reports of their effects in other cancers have been contradictory, in large part due to their pleiotropic and sometimes opposing effects on cell viability and apoptosis, metastasis, angiogenesis, tissue remodeling and EMT [37]. Because kallikreins often act in a cascade and at least 12 kallikreins are concomitantly upregulated in ovarian cancer it is difficult to parse the individual contribution of each kallikrein to the pathophysiology of this disease.

To systematically investigate the contributions of KLK5, 6 and 10 to ovarian cancer development, the ES-2 cell line was used, since it did not express any of the kallikreins tested and readily forms tumours in nude mice. The ES-2 ovarian cancer cell line was originally derived from a patient with a clear cell tumour [53], however when xenografted it is known to make undifferentiated tumours [39]. From this cell line we generated clones overexpressing KLK5, 6 and 10 alone or in pairs. The resulting clones displayed altered anchorage-independent growth *in vitro*, as well as varying aggressivity *in vivo* as measured by survival of xenografted nude mice. Cells overexpressing KLK5, 5/6, 5/10, and 6/10 produced significantly fewer colonies in soft agar than vector-transfected controls. Similarly, mice xenografted with cells overexpressing KLK10, 5/6, 5/10, 6/10 had a significant survival advantage over their respective control mice, while mice with KLK6-secreting tumours had significantly decreased survival. The increased survival of the KLK10 group was reminiscent of the decreased tumourigenicity of the MDA-MB-231 breast cancer cell line overexpressing KLK10 observed by Goyal et al [44]. This observation further supports the hypothesis of KLK10 as a putative tumour suppressor, silenced in prostate, testicular, and breast cancer as well as in acute lymphoblastic leukemia. Furthermore, it may be that the ES-2 cell line is exquisitely sensitive to overexpression of KLK10 since, in these cells, the KLK10 locus is hypermethylated, suggesting that silencing contributed to its transformation [36]. In contrast to the KLK10 group, the mice xenografted with cells overexpressing KLK6 died significantly earlier than the control mice. The increased aggressiveness of the KLK6 clone was not unexpected as KLK6 overexpression is thought to be an early phenomenon in ovarian carcinoma development [25]. KLK6 has been associated with increased invasiveness, growth and angiogenesis, by virtue of its ability to degrade ECM components such as denatured type I collagen, fibronectin, vitronectin and laminin [54], or activate PAR-2 signaling [55] which has been implicated in mediating cellular proliferation in colon cancer cells [56].

The importance of the specific mix of kallikreins present and their relative abundance on the activome is underscored by the drastic difference within our clones, and with other published reports such as the findings of Prezas et al. [57] who have shown that the OV-MZ-6 ovarian cancer cell line engineered to co-express KLK4/5/6/7 displayed an increased tumourigenicity. Furthermore, the data suggests that some kallikreins may have dominant or inactivating/activating effects over other kallikreins, suggesting for example that the drastically different behaviour of KLK6 versus KLK5/6 clones, could be due to the ability of KLK5 to inactivate other kallikreins *in vitro* [58]. Conversely animals with tumours expressing a combination of KLK6/10 behave differently than their single expressing counterparts suggesting that the kallikreins can interact. The basis of this interaction may rely on the ability of KLK6 to cleave and activate itself [29], while KLK10's function may be independent of enzymatic activity since it appears to be catalytically inactive *in vivo* [52]. Finally, the relative abundance of serum kallikreins as measured in the xenografted mice prior to endpoint suggest that dosage may play an important role in the phenotype: the high concentration of KLK10 in both the single expresser and the KLK6/10 group may explain the strong protective effect of KLK10. Taken together these results suggest that kallikreins 5, 6 and 10 can mediate effects important for tumourigenicity, and their interactions may be complex and dependent on the kallikreins' activome and the relative abundance of the various kallikreins.

To understand the mechanisms underlying the survival differences, it was possible to exploit the fact that the implanted tumours secreted kallikreins into the blood and ascites, thus providing us with a means to track tumour burden. The use of kallikreins to track tumour burden has previously been documented in the clinic, most notably with KLK3 (PSA) in prostate cancer [59]–[61]. It has also been suggested that both KLK6 and KLK10 could be useful diagnostic biomarkers, which, combined with CA-125 can increase the sensitivity of the screening test [22]. Similarly we detected kallikreins 5, 6, and 10 in the circulation well before the onset of any symptoms, and their levels increased as disease progressed, only to peak at necropsy. Interestingly, the disease-free surviving mice of groups KLK5, KLK5/6, KLK5/10 and KLK6/10, never displayed any detectable levels of kallikreins, suggesting a failure to implant or to grow to a detectable size. From these combined data, it is possible to infer that some of the survival effects of kallikreins 5, 6 and 10 are mediated by an inhibition of tumour implantation, possibly because of inhibition of anchorage-independent growth.

The finding that the disease phenotype also varied qualitatively amongst groups gave insights into the contribution of kallikreins to the pathophysiology of ovarian cancer. The most common endpoint in the survival experiment was distension as a result of ascites, therefore, a reduction in the incidence of ascites can have a large influence on survival despite rarely causing mortality in patients. As such, groups KLK5/10 and KLK6/10 had a marked reduction in the incidence of ascites and a corresponding longer survival. While the mechanisms by which KLK5, 6 and 10 influence ascites formation has not yet been established, kallikreins are known to mediate processes such as inflammation, oedema, angiogenesis and blood pressure [2], all of which are relevant to ascites accumulation [56], [62], [63]. Furthermore all groups which had a survival advantage had a marked reduction in the incidence of aggregates in the ascites, possibly because the effect of kallikreins on anchorage-independent growth. Aggregates in the ascites of ovarian cancer patients have previously been identified, and may contribute to the spreading of the disease [64]. Taken together, these results raise the possibility that KLK5, 6 and 10 play an inhibitory role in the formation of ascites and the cellular aggregates within it, which consequently reduces the morbidity and mortality of the mice. While tumour burden, ascites volume, or sites of metastasis do not generally differ at endpoint, this is an artifact of the endpoints used in this study since animals reaching a common set of criteria before being sacrificed does not reflect differences in the rate of progression of these attributes.

Paradoxically, all three kallikreins tested are already known to be elevated in ascites of patients [46], [52], particularly KLK6, albeit at lower levels than what was recorded in the ascites of mice in this study. It is tempting to speculate that patients with high levels of circulating KLK10, particularly in combination with low or null amounts of KLK6, may be less prone to ascites accumulation and those ascites may be less likely to contain cellular aggregates.

The positive and dominant effects of KLK10 on overall survival make it an attractive putative therapeutic agent for ovarian cancer. To test this prospect, a recombinant protein was generated, which was found to be devoid of proteolytic activity, in accordance with earlier published studies [22]. The recombinant KLK10 protein was injected into the peritoneum to maximize the exposure of peritoneal tumours and detached cellular aggregates to the drug. The recombinant KLK10 was well tolerated at up to 5 mg, although only a fraction of the drug was detected in the circulation, and it was quickly cleared from the blood. Remarkably, the recombinant KLK10, despite being present only intermittently, was sufficient to significantly increase survival of treated mice at doses of 5 mg once or twice daily, and the study concluded with one mouse without detectable disease. While we do not know whether the cells failed to implant in the presence of KLK10 at the time of injection, or the tumours regressed later during the treatment, the substrate-dependent growth of the clones suggest the former.

The molecular pathway by which the catalytically inactive KLK10 exerts its biological effects remains elusive, despite the accumulating evidence of its tumour-suppressing qualities. *In vitro* results suggest KLK10 may be cytotoxic to cancer cells at high concentrations and that a component of fetal calf serum can inhibit this toxicity. It is unclear how KLK10 may mediate its anti-tumour effects, however the absence of toxicity in mice and the potent in-vitro response to the recombinant KLK10 peptide suggest a promising therapeutic window. Taken together these results indicate that the effects observed with the KLK10 secreting clones on survival and on the pathophysiology are specific to KLK10 and could be partially recapitulated with a recombinant protein, suggesting it may have therapeutic value. Finally these findings support the hypothesis that KLK10 is a tumour suppressor and further underline the involvement of KLK5, 6 and 10 in ovarian pathophysiology.

## Supporting Information

Figure S1 **Ascites volume and tumour burden of xenografted mice treated with recombinant KLK10.** A) The ascites volume was measured at endpoint in mice treated with either PBS or one or two doses of recombinant KLK10 per day. B) tumour burden, as defined as total mass or excisable tumour at endpoint was recorded in the same treatment groups.
(EPS)

Table S1 **KLK5, 6, 8, 10, 13 and 14 concentrations, in the media at 72 h, in a panel of 13 ovarian cancer cell lines.**
(DOCX)
