# Supplementary material for: Kallikreins 5, 6 and 10 Differentially Alter Pathophysiology and Overall Survival in an Ovarian Cancer Xenograft Model
Source: PLoS One. 2011 Nov 15;6(11):e26075. doi: 10.1371/journal.pone.0026075 (PMC3216928; doi:10.1371/journal.pone.0026075)
Supplement: Table S1 — KLK5, 6, 8, 10, 13 and 14 concentrations, in the media at 72 h, in a panel of 13 ovarian cancer cell lines. (DOCX) [file pone.0026075.s002.docx]

**Supplemental table 1. KLK5, 6, 8, 10, 13 and 14 concentrations, ion the media at 72h, in a panel of 13 ovarian cancer cell lines.**

| Cell lines | Kallikrein concentration in media (ng/ml) | | | | | |
| --- | --- | --- | --- | --- | --- | --- |
|  | KLK5 | KLK6 | KLK8 | KLK10 | KLK13 | KLK14 |
| CAOV-3 | 13.8±3.2 | 6.0±2.3 | 0.4±0.5 | 4.3±4 | ND | ND |
| OVCAR-3 | 4.2±0.7 | 2.1±0.5 | 2.2±0.5 | 1±0.9 | ND | ND |
| OVCAR-4 | 0.4±0.2 | 3.5±1.7 | 0.5±0.2 | 3.9±1.3 | 0.1±0 | ND |
| OV2008 | 18.4±2.6 | 3.4±0.3 | 4.5±1.1 | 27.9±18.9 | 7.5±2.8 | ND |
| C13 | 6.6±1 | 6.7±5.4 | ND | 29.5±8.4 | 1.4±0.3 | ND |
| OVCA433 | 15.1±2.1 | 24.3±5 | ND | ND | ND | ND |
| SKOV-3 | ND | ND | ND | ND | ND | ND |
| OVCA429 | ND | ND | ND | ND | ND | ND |
| ES-2 | ND | ND | ND | ND | ND | ND |
| HEY | ND | ND | ND | ND | ND | ND |
| A2780cp | ND | ND | ND | ND | ND | ND |
| A2780s | ND | ND | ND | ND | ND | ND |
| OCC-1 | ND | ND | ND | ND | ND | ND |

ND=not detectable. Values represent the mean concentration from 3 ELISA experiements ± SEM.
